# Dual‐Crosslinked Dynamic Hydrogel Incorporating {Mo_154_} with pH and NIR Responsiveness for Chemo‐Photothermal Therapy

**DOI:** 10.1002/adma.202007761

**Published:** 2021-08-11

**Authors:** Gabriela Guedes, Shiqi Wang, Flavia Fontana, Patrícia Figueiredo, Jere Lindén, Alexandra Correia, Ricardo J. B. Pinto, Sami Hietala, Filipa L. Sousa, Hélder A. Santos

**Affiliations:** ^1^ Drug Research Program Division of Pharmaceutical Chemistry and Technology Faculty of Pharmacy University of Helsinki Helsinki FI‐00014 Finland; ^2^ Chemistry Department University of Aveiro Campus Universitário de Santiago Aveiro 3810‐193 Portugal; ^3^ CICECO‐Aveiro Institute of Materials University of Aveiro Campus Universitário de Santiago Aveiro 3810‐193 Portugal; ^4^ Faculty of Veterinary Medicine Finnish Centre for Laboratory Animal Pathology (FCLAP)/HiLIFE University of Helsinki Helsinki FI‐00014 Finland; ^5^ Department of Chemistry University of Helsinki Helsinki FI‐00014 Finland; ^6^ Helsinki Institute of Life Science (HiLIFE) University of Helsinki Helsinki FI‐00014 Finland

**Keywords:** {Mo _154_}, chemo‐photothermal therapy, injectable hydrogel, NIR‐responsive, pH‐responsive

## Abstract

Polyoxometalates are an emerging class of molecular clusters, with well‐defined structures and chemical compositions that are produced through simple, low‐cost, and highly reproducible methods. In particular, the wheel‐shaped cluster {Mo_154_} is a promising photothermal agent due to its intervalence charge transfer transitions. However, its toxicity hinders its systemic administration, being the development of a localized delivery system still incipient. Herein, an injectable and self‐healing hydrogel of easy preparation and administration is developed, incorporating both {Mo_154_} and doxorubicin for synergistic photothermal and chemotherapy applications. The hydrogel is composed of benzylaldehyde functionalized polyethylene glycol, poly(*N*‐isopropylacrylamide) functionalized chitosan and {Mo_154_}. The gelation occurs within 60 s at room temperature, and the dual crosslinking by Schiff base and electrostatic interactions generates a dynamic network, which enables self‐healing after injection. Moreover, the hydrogel delivers chemotherapeutic drugs, with a release triggered by dual near infra‐red (NIR) radiation and pH changes. This stimuli‐responsive release system along with the photothermal conversion ability of the hydrogel allows the simultaneous combination of photothermal and chemotherapy. This synergic system efficiently ablates the cancer tumor in vivo with no systemic toxicity. Overall, this work paves the way for the development of novel {Mo_154_}‐based systems, incorporated in self‐healing and injectable hydrogels for dual chemo‐photothermal therapy.

## Graphical abstract

A dual pH‐ and near infra‐red (NIR)‐responsive hydrogel is developed composed of benzylaldehyde functionalized poly(ethylene glycol), poly(*N*‐isopropylacrylamide) functionalized chitosan, and a polyoxometalate {Mo_154_}. Crosslinked by Schiff base and electrostatic interactions, the hydrogel has dynamic networks, injectability, and fast self‐healing properties. Combining doxorubicin and NIR laser irradiation, the hydrogel shows thermal ablation of cancer cells and simultaneous release of drugs for chemo‐photothermal therapy.

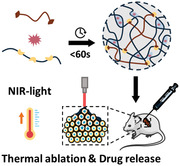

## 1 Introduction

Injectable hydrogels have shown great prospects in recent biomedical applications, due to their minimally invasive character, high accessibility to parts of the body that usually are hard to reach, and the ability to conform to any shape.^[^ ^1^ ^]^ In addition, injectable hydrogels also exhibit the conventional advantages of hydrogels, such as permeability to oxygen and nutrients, biocompatibility, and porous structure that allows the loading of therapeutic agents.^[^ ^2^, ^3^ ^]^ The most used strategy to prepare injectable hydrogels is the injection of liquid precursors, followed by the subsequent gelation in situ.^[^ ^4^ ^]^ However, there is an inherent difficulty to control the gelation time in vivo.^[^ ^5^, ^6^ ^]^ Slow gelation can culminate in the spreading of the hydrogel or its components, possibly leading to the loss of the cargo molecules and the leakage of toxic liquid precursors, while too fast gelation can originate a nonuniform hydrogel or the premature gelation in the syringe leading to needle blocking.^[^ ^5^ ^]^ To overcome these limitations, self‐healing and shear‐thinning hydrogels have been explored.^[^ ^7^ ^]^ These type of hydrogels enable better encapsulation of the cargo molecules because the hydrogel is already gelled when administrated.^[^ ^8^ ^]^ Therefore, many rapid self‐healing injectable hydrogels have been developed to deliver drugs, bioactive molecules, and cells for tissue regeneration and repair, therapies for infectious and inflammatory diseases, and cancer.^[^ ^2^, ^9^ ^]^ Interestingly, hydrogels can be tuned to respond to both endogenous and exogenous stimuli, such as pH, temperature, and light.^[^ ^1^ ^]^ Such environmental responsiveness has been widely used to deliver therapeutic cargos in a controllable manner.^[^ ^10^ ^]^ For example, the natural difference regarding the pH of the healthy (≈7.4) and cancer tissues (ranging from 6.5 to 7.2),^[^ ^11^ ^]^ triggers the drug release from pH‐responsive hydrogels either by swelling^[^ ^12^ ^]^ or hydrogel degradation.^[^ ^13^ ^]^ Another common stimulus is near infra‐red (NIR) light, due to the non‐invasiveness and to the possibility of triggering the drug release in an accurate spatiotemporal manner.^[^ ^14^ ^]^ In addition to acting as a stimulus, NIR can also thermally ablate cancer cells in a treatment known as photothermal therapy (PTT).^[^ ^15^ ^]^ PTT uses photothermal agents to transduce the absorbed light (photon energy) into heat (thermal energy) and induce hyperthermia.^[^ ^16^ ^]^ More than a treatment per se, hyperthermia is used as an adjuvant for common therapies due to the induced thermo‐sensitization effects (e.g., inhibition of DNA synthesis and repair, cell membrane, and cytoskeleton damage), creating a synergy between hyperthermia and other anticancer therapies, such as gene therapy radiotherapy and chemotherapy.^[^ ^17^, ^18^, ^19^, ^20^ ^]^ Additionally, the temperature increase caused by PTT promotes the accumulation and penetration of drugs in the tumor.^[^ ^21^, ^22^ ^]^ As a highly effective, selective, simple, and minimally invasive treatment able to eliminate several types of cancers,^[^ ^23^, ^24^ ^]^ PTT has been emerging to overcome the harsh side effects and maximize the treatment outcomes of the cancer therapies currently used.^[^ ^25^ ^]^

Polyoxometalates (POMs) are molecular clusters composed of early transition metals and oxygen, which can incorporate a great diversity of other elements and molecules, giving rise to structural diversity, as well as a vast panoply of properties.^[^ ^26^ ^]^ Recently, it was found that some POMs (e.g., [GdW_10_O_36_]^9−^, [PMo_12_O_40_]^3−^, [PMo_2_W_10_O_40_]^5−^, and [Mo^VI^_126_Mo^V^_28_O_462_H_14_(H_2_O)_70_]_0.5_[Mo^VI^_124_Mo^V^_28_O_457_H_14_(H_2_O)_68_]_0.5_]^15−^≡{Mo_154_}) present intervalence charge transfer (IVCT) transitions between metal ions leading to strong absorptions in the NIR region.^[^ ^27^, ^28^, ^29^, ^30^ ^]^ The IVCT transitions impart these POMs with promising photothermal conversion ability, making them an emerging class of PTT agents.^[^ ^31^, ^32^, ^33^, ^34^, ^35^, ^36^ ^]^ In particular, {Mo_154_} anion, a giant polyoxomolybdate with a very well defined chemical and structural composition and 3.4 nm of diameter, exhibited a superior photothermal conversion efficiency (30.9%)^[^ ^37^ ^]^ comparable to or higher than common photothermal agents, such as gold nanoparticles of different morphologies (13–21%).^[^ ^38^ ^]^ Moreover, their simple and highly reproducible synthesis allows fast and efficient production at low‐cost.^[^ ^39^, ^40^ ^]^ Despite all these advantages, the systemic administration of POMs is still hindered due to its toxicity, lack of selectivity, and low bioavailability.^[^ ^41^ ^]^ For this reason, the encapsulation of POMs in a hydrogel could be a simple and effective strategy to overcome this issue. In recent years, only very few pioneer works studied the incorporation of POMs in hydrogels for its possible biomedical applications, mostly focusing on material development, including mechanical behavior, physicochemical properties, and drug release potentials.^[^ ^42^, ^43^, ^44^, ^45^ ^]^ However, to the best of our knowledge, there is no study reporting POMs containing hydrogels for PTT‐related applications.

**Figure 1:**
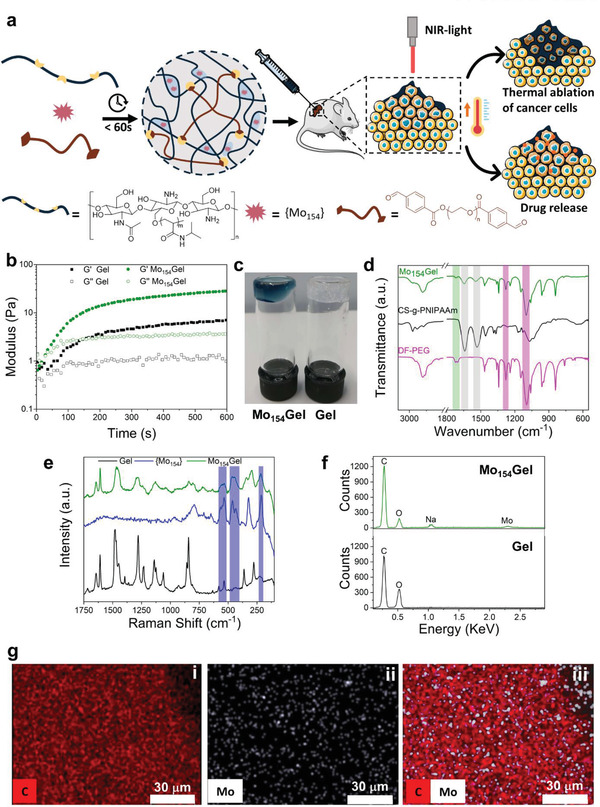
Preparation and characterization of Mo_154_Gel. a) General scheme showing the design, preparation, and synergistic effects of PTT and NIR‐triggered DOX release from Mo_154_Gel. Mouse, syringe, and tumor cell images are from SERVIER MEDICAL ART under the Creative Commons Attribution 3.0 License. b) Time sweep rheological analysis of the Gel (black) and Mo_154_Gel (green) performed at 25 °C and 1% of strain. c) Photo of the Mo_154_Gel and Gel. d) FTIR spectra of DF‐PEG, CS‐g‐PNIPAAm, and Mo_154_Gel. e) Solid‐state Raman spectra of Gel, {Mo_154_}, and Mo_154_Gel (λ_e_ = 1064 nm). f) Energy‐dispersive X‐ray (EDS) spectra of Mo_154_Gel and Gel with the respective assignments of the elements detected. g) EDS‐mapping micrographs of Mo_154_Gel: i) carbon, ii) molybdenum, and iii) carbon and molybdenum merged maps.

In the present work, we incorporated a POM, namely the anion {Mo_154_}, in an injectable hydrogel, where it plays a dual role, behaving both as a cross‐linker (through electrostatic interactions) and as a photothermal transducer, that allows the simultaneous PTT and NIR‐triggered chemotherapy drug release. In this system, we chose a di‐benzaldehyde functionalized polyethylene glycol (DF‐PEG) and a thermo‐responsive derivative from chitosan (CS‐g‐PNIPAAm) to generate the hydrogel network due to their good biocompatibility^[^ ^46^, ^47^, ^48^, ^49^ ^]^ and abundance.^[^ ^12^ ^]^ Moreover, CS‐g‐PNIPAAm imparted the hydrogel with thermo‐responsiveness, allowing the drug release at elevated temperature after laser irradiation. The hydrogel was formed by the dynamic imine bond between the aldehyde from DF‐PEG and the amine from CS‐g‐PNIPAAm, as well as the dynamic electrostatic interactions between the anionic {Mo_154_} and the cationic CS‐g‐PNIPAAm (**Figure** **1**a). The double dynamic network enables fast self‐healing properties by rearranging the cleaved bonds after injection. Furthermore, the imine bond is pH‐sensitive, which allows faster and more complete drug release in acidic environment. By rational design of the POM‐containing hydrogel, we aimed to combine the synergistic effects from PTT and chemotherapy, to maximize their therapeutic efficacy in cancer treatment.

## 2 Results and Discussion

The polymers used in this study (CS‐g‐PNIPAAm and DF‐PEG) were synthesized according to literature reports.^[^ ^50^, ^51^ ^]^ The structures of both polymers were characterized by ^1^H‐NMR (Figures S1,S2, Supporting Information), and the results were in good agreement with literature.^[^ ^50^, ^51^ ^]^ The thermo‐responsiveness of CS‐g‐PNIPAAm was characterized by differential scanning calorimetry (DSC), and the lower critical solution temperature (LCST) is 31 °C.

Then, the hydrogel (named as Mo_154_Gel) was developed by simple mixing solutions of CS‐g‐PNIPAAm, DF‐PEG, and {Mo_154_} at room temperature (Figure 1a). The hydrogel formulation had a composition of 1.5 wt% Cs‐g‐PNIPAAm, 5.5 wt% DF‐PEG (benzaldehyde:amine = 1:3), and 0.092 wt% {Mo_154_}. The excessive number of amine groups compared with benzaldehyde, allows for {Mo_154_} interaction via electrostatic interactions. A hydrogel control without POM (named as Gel) was also prepared in the same manner, to study the role of {Mo_154_} in the hydrogel cross‐linking network. As verified by the time sweep rheological analysis (Figure 1b) and by inversion test (Figure 1c), both hydrogels formed within 50 s after simply mixing all components by vortex. The storage modulus (*G′*) and loss modulus (*G″*) of Mo_154_Gel were higher than those of Gel, indicating that the {Mo_154_} anion contributed to the hydrogel cross‐linking, possibly through the formation of electrostatic bonds between the positively charged amine groups on chitosan backbone and the highly negative charged {Mo_154_}.^[^ ^44^ ^]^

The chemical composition of Mo_154_Gel was analyzed using Fourier transform infrared spectroscopy (FTIR) and Raman spectroscopy (Figure 1d,e). The bands highlighted in Figure 1d confirmed the presence of the CS‐g‐PNIPAAm's (in grey shade: at 1639 and 1533 cm^−1^ ascribed to the amide I and II,^[^ ^52^, ^53^ ^]^ respectively, from PNIPAAm in CS‐g‐PNIPAAm) and DF‐PEG's (in pink shade: at 1341 cm^−1^ attributed to the C—H bending, and at 1095 cm^−1^ associated with C—O stretching characteristic from PEG)^[^ ^54^, ^55^ ^]^ functional groups in Mo_154_Gel.^[^ ^52^, ^53^, ^54^ ^]^ Moreover, the decrease in the intensity of the band at 1712 cm^−1^ (in green shade), is assigned to the aldehyde and ester carbonyls of the DF‐PEG,^[^ ^51^ ^]^ confirming the polymers’ cross‐linking by the formation of Schiff base bonds among the benzaldehyde groups on DF‐PEG and the amine groups on the CS backbone of CS‐g‐PNIPAAm. Additionally, the Raman spectrum (Figure 1e) of Mo_154_Gel showed the fingerprint of {Mo_154_} (in blue shade),^[^ ^56^ ^]^ proving its presence and integrity in the prepared hydrogel.

Through energy‐dispersive X‐ray spectroscopy (EDS) analysis (Figure 1g), the appearance of the Mo signal in Mo_154_Gel was observed. This indicates, along with the FTIR and Raman data, that Mo was successfully introduced in the hydrogel in the form of {Mo_154_}. Moreover, EDS mapping images (Figure 1h) showed that the POM was evenly distributed in the Mo_154_Gel.

**Figure 2:**
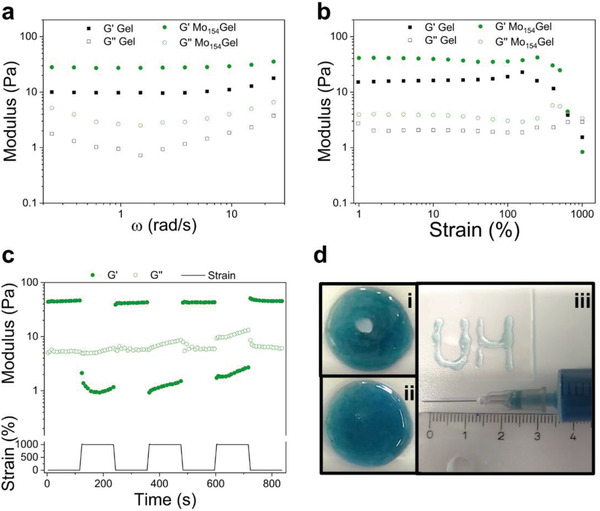
a) Frequency sweep of Gel and Mo_154_Gel. The assay was performed at a constant strain of 1% and 25 °C. b) Strain sweep of Gel and Mo_154_Gel. The assay was performed at a constant frequency of 1 Hz and 25 °C. c) The *G′* and *G″* in continuous step strain measurements (cycles of 1% and 1000% of strain at 4 min for each cycle). d) Self‐healing and injectability: photo of the hydrogel i) right after punching a hole (≈0.8 cm in diameter) and ii) 4 min after. iii) Photo of the hydrogel extruded using a syringe to write letters “UH.”

The rheological behavior of Mo_154_Gel was studied through frequency sweep (**Figure** **2**a), strain sweep (Figure 2b), and temperature ramp sweep (Figure S4, Supporting Information) measurements. The *G″* variation observed in the frequency sweep tests (Figure 2a) is typical for hydrogel networks formed by dynamic cross‐links instead of rigid covalent bonds.^[^ ^57^, ^58^ ^]^ This agrees with the FTIR data in Figure 1d since both Schiff base and electrostatic interactions are dynamic bonds. The dynamic cross‐linking is fundamental for the self‐healing ability of a hydrogel because it allows the bond formation after break or damage.^[^ ^9^ ^]^ The temperature ramp (Figure S3, Supporting Information) suggests that the storage modulus (*G*′) was stable from 15 to 30 °C, with a slight increase until 40 °C. The slight increase in *G*′ after 30 °C is probably due to the thermo‐responsive conformational change from chitosan‐g‐PNIPAAm. The PNIPAAm chains aggregate when temperature is higher than LCST (31 °C), and the aggregation makes the gel a bit stiffer.

To evaluate the self‐healing behavior of the hydrogel, a step strain measurement was carried out by applying a strain (1000%), higher than the limit of the linear viscosity region (LVR) (Figure 2b), for 2 min, followed by the application of a low strain within LVR (1%) in 4 cycles (Figure 2c). With the increase of the strain from 1% to 1000%, the storage modulus decreased from around 45 to 1 Pa due to the breakdown of the hydrogel network. However, when the high strain (1000%) was removed, both *G*′ and *G*″ exhibited total recovery within few seconds in all cycles. The fast recovery of Mo_154_Gel is explained by the reversible behavior of the imine and electrostatic bonds. These types of bonds are highly reversible and act as sacrificial bonds breaking at high stress, but readily reform after its removal.^[^ ^59^ ^]^ Therefore, the dynamic equilibrium exhibited by both the Schiff base and electrostatic bonds helps the quick stabilization of the hydrogel network under stress and allows the fast recovery (healing) of the hydrogel.^[^ ^59^ ^]^

To further evaluate the self‐healing ability of the prepared hydrogel, a macroscopic self‐healing test was performed by punching a hole in a freshly prepared hydrogel (Figure 2d‐i). After 4 min at room temperature (Figure 2d‐ii; Video S1, Supporting Information), the hole was completely closed, indicating an efficient self‐healing and, consequently, the repair of the cross‐linked network.

The injectability of Mo_154_Gel was further tested by extruding it through a 22G syringe needle (0.7 mm in diameter). The letters “UH” were successfully written using the hydrogel, showing a uniform structure (Figure 2d‐iii). The shear thinning properties were also studied by a shear flow sweep assay (Figure S5, Supporting Information), showing that the viscosity of Mo_154_Gel decreased steadily as the shear rate increased from 1 to 100 s^−1^. Both the injection and the flow sweep test confirm the good injectability of the prepared hydrogel. This behavior indicated that the developed hydrogel could be implanted through a minimally invasive injection.

**Figure 3:**
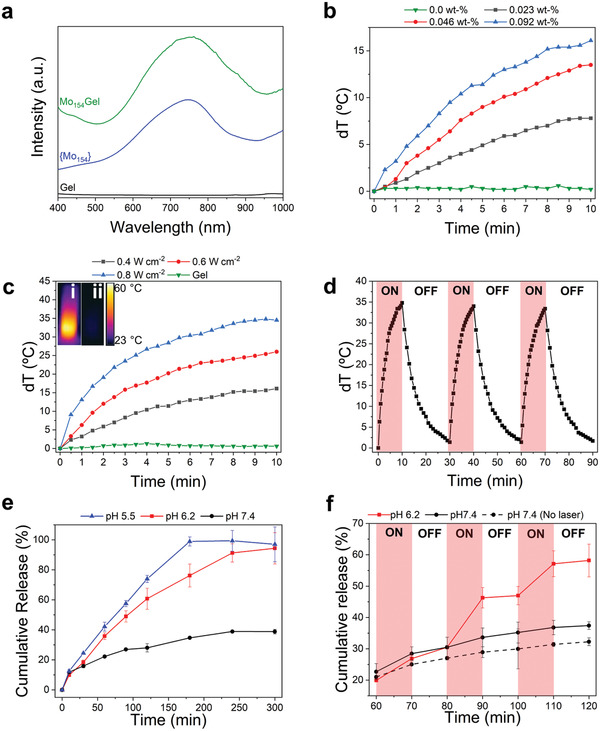
a) UV–vis spectra of Mo_154_Gel, Gel, and {Mo_154_} aqueous solution. Photothermal conversion curves of b) Mo_154_Gel with different {Mo_154_} concentrations (0, 0.023, 0.046, 0.092 wt%) irradiated for 10 min (0.4 W cm^−2^) and c) Mo_154_Gel (0.092 wt% {Mo_154_}) exposed to different irradiation powers. The inset exhibits the IR thermal images showing the temperature of the i) Mo_154_Gel and ii) Gel after 10 min of NIR irradiation (0.8 W cm^−2^). d) Photothermal stability of Mo_154_Gel under cycles of irradiation of 10 min ON (0.8 W cm^−2^) and 20 min OFF. e) The pH‐responsive release of DOX (pH 5.5, 6.2, and 7.4 at 37 °C) from Mo_154_Gel. f) The NIR laser (0.8 W cm^−2^) triggered release of DOX from Mo_154_Gel at pH 7.4 and 6.2 at 37 °C. The dashed line corresponds to the release in the absence of the laser cycles (pH 7.4). Results are presented as mean ± standard deviation (*N* = 3).

After characterizing the physicochemical behavior of Mo_154_Gel, we investigated its potential as a PTT agent. The UV–vis absorption spectrum of Mo_154_Gel shows a significant band in the NIR region (**Figure** **3**a), which is characteristic of the {Mo_154_}, and that arises from IVCT between the delocalized electrons in the Mo^V^ and Mo^VI^ centers.^[^ ^60^ ^]^ The maximum of the absorption band is centered at 745 nm, indicating that the first NIR window wavelengths usually used for PTT (around 808 nm) are applicable to Mo_154_Gel. The strong absorbance in the NIR region imparted by the presence of the POM in the hydrogel is essential to confer photothermal conversion ability to the formulation.

Next, motivated by the notable absorbance of Mo_154_Gel in the NIR region, we decided to investigate its photothermal conversion ability. First, Gel and Mo_154_Gel with different {Mo_154_} concentrations (0.023, 0.046, 0.092 wt%) were irradiated with a laser (808 nm, 0.4 W cm^−2^) at room temperature (≈25 °C) (Figure 3b). As expected, the temperature increase (dT) showed a strong dependency on the {Mo_154_} concentration: for a 0.023 wt% concentration, dT was 8 °C, increasing to almost 12 °C when the concentration was doubled to 0.046 wt%. A further increase in the {Mo_154_} concentration to 0.092 wt% made dT over 15 °C, which is promising to induce in vivo hyperthermia.^[^ ^61^ ^]^ When increasing the irradiation power to 0.6 and 0.8 W cm^−2^ (Figure 3c), higher dT were achieved, respectively, 26.0 and 34.5 °C. As shown in the IR thermal images (Figure S6, Supporting Information, and the inset of Figure 3c), Gel had almost no temperature change after laser irradiation (0.8 W cm^−2^), while Mo_154_Gel showed a rapid temperature increase to 57.8 °C after 10 min. It is noteworthy that the irradiance used in this assay (0.8 W cm^−2^) to reach a total temperature increase of ≈35 °C, is lower than those being described in the literature (mostly between 1.0 and 5.8 W cm^−2^) for the same temperature increase (≈30 °C).^[^ ^62^, ^63^, ^64^ ^]^ Therefore, to have a good compromise between the amount the {Mo_154_}, power density, and the temperature increase needed to effectively ablate the tumor, we chose the formulation with 0.092 wt% of {Mo_154_} and the power density of 0.8 W cm^−2^ for the subsequent experiments. Additionally, the hydrogel was exposed to three ON (10 min, 0.8 W cm^−2^) and OFF (20 min) cycles of laser irradiation (Figure 3d). Throughout repeated exposures to irradiation, no evident change was observed in the temperature increase and a plateau (dT around 35 °C) was reached, thus indicating the good photothermal stability of the hydrogel.

To further evaluate the possibility of using Mo_154_Gel in drug delivery applications, we studied the loading and release of DOX, used as model anticancer drug. Unlike the regular nanocarriers with a relatively low drug loading efficiency, hydrogels can completely load the drugs without any further purification.^[^ ^65^ ^]^ In this case, DOX was added before the gelation of the Mo_154_Gel, and after the gelation, all the cargo was loaded in the injectable hydrogel (Mo_154_GelDOX). The release profile was studied at three different pH‐values, 5.5, 6.2, and 7.4, mimicking the mild acidic environment of late stage tumors, early stage tumors, and the physiological environment, respectively.^[^ ^66^ ^]^ Mo_154_GelDOX showed a pH‐responsive release behavior, that is faster and more complete at pH 5.5 or 6.2 than at pH 7.4 (Figure 3e). At pH 5.5 and 6.2, Mo_154_GelDOX showed an initial burst release in the first hour, which is probably attributed to the rapid release of the weakly attached drugs on the outer surface of the hydrogel. At pH 6.2, after 240 min, the drug release was almost complete (up to 91%), while at pH 5.5 this happened even earlier (in the 180 min time point). In contrast, at pH 7.4 after 300 min the release was only around 40%. Therefore, the acidic environment favored the drug release profile, enabling the hydrogel as a pH‐dependent drug delivery system.

In addition to the pH‐dependent release, Mo_154_Gel is also expected to have a NIR‐triggered release, since we previously proved the photothermal conversion of the hydrogel (Figure 3b–d) and the presence of a thermo‐responsive copolymer (CS‐g‐PNIPAAm) in the hydrogel constitution (Figures 1d). To verify this hypothesis, we studied the release of DOX from Mo_154_GelDOX in 3 cycles of ON/OFF laser irradiation at pH 6.2 and pH 7.4 (Figure 3f). All the hydrogel samples were equilibrated in pH 7.4 buffer for 1 h before the laser‐irradiation, to eliminate the burst release effect on the final results. At pH 7.4, NIR‐light irradiation only slightly increased the release by 5%, compared with the sample without NIR irradiation. However, when the hydrogel was irradiated at pH 6.2, there was a significant increase in the release rate by 26% at the end of all cycles. In the first cycle, Mo_154_GelDOX showed similar release behavior compared with pH 7.4 sample, probably because of the ongoing buffer exchange. Then, in the second and third cycle, the release rate significantly increased with laser irradiation and immediately slowed down when the laser was OFF, indicating the photo‐thermal responsiveness of the hydrogel. The data confirms the dual‐responsiveness of the hydrogel and the drug release triggered by both acidic pH and NIR light, since it shows that only in the presence of both stimuli there is a significant increase in the release.

**Figure 4:**
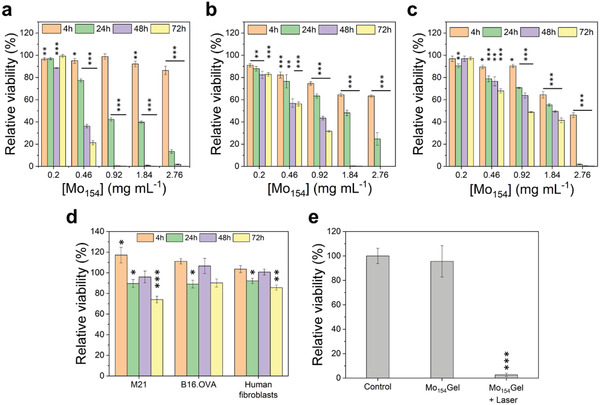
The relative cell viability of a) M21, b) B16.OVA, and c) primary human fibroblast cells after the exposure of {Mo_154_} for 4, 24, 48, and 72 h. The results were plotted as mean ± standard deviation (*N* = 4). d) The relative cell viability of M21, B16.OVA, and primary human fibroblast cells after the exposure of Mo_154_Gel for 4, 24, 48, and 72 h. The results were plotted as mean ± standard deviation (*N* = 3). e) The relative viability of M21 cells in the presence of Mo_154_Gel in the dark, or exposed to 10 min of laser irradiation (0.8 W cm^−2^). The results were plotted as mean ± standard deviation (*N* = 4). The statistical analysis was performed using One‐way ANOVA (*denotes significant differences with a significance level of *p* < 0.05, **p < 0.01, and ***p < 0.001), by comparing each group with the corresponding negative control at the same timepoint.

Inspired by the dual pH‐ and NIR‐responsive release profile and the PTT behavior of the hydrogel, we proceeded with biological tests. First, we evaluated the cytotoxicity of {Mo_154_} and whether it affects the ATP production on human melanoma cells (M21), murine melanoma cells (B16.OVA), and primary human fibroblast cells (**Figure** **4**a–c, respectively), by Cell‐Titer Glo luminescent assay. As expected, {Mo_154_} interfered with ATP production in a concentration and time‐dependent manner. In particular, when the concentration of {Mo_154_} was 0.92 mg mL^−1^, concentration equivalent to the one used in the Mo_154_Gel, almost all M21 cells lost their ATP production capability after 48 h incubation. B16.OVA and human fibroblasts were more resistant to {Mo_154_}, but their ATP production also significantly decreased after 48 h. After 72 h incubation with {Mo_154_} at 0.92 mg mL^−1^, the ATP produced by B16.OVA and human fibroblasts further decreased to 32% and 49%, respectively. The incorporation of {Mo_154_} in the hydrogel significantly improved the relative cell viability for all the tested cell lines and time‐points (Figure 4d), allowing the use of a {Mo_154_} concentration for PTT able to induce a suitable temperature increase with minimal ATP‐production inhibitory effects. Then, we evaluated whether the PTT would eliminate M21 melanoma cells in vitro. The cells were exposed to the injected Mo_154_Gel with 10 min laser irradiation or without irradiation (Figure 4e). Without irradiation and in the presence of injected Mo_154_Gel, the cell viability remained very close to 100%, indicating the absence of toxicity of the hydrogel, in agreement with the results showed in Figure 4d. However, when the cells were exposed to Mo_154_Gel and further irradiated with NIR laser (808 nm, 0.8 W cm^−2^), there was a very significant decrease in the cell viability to around 2%, indicating the potency of PTT in cancer cell ablation. The high temperature caused by the photothermal conversion of the Mo_154_Gel upon laser irradiation is potentially harmful for all cell types, including healthy cells. However, the localized character of PTT and the confined location of Mo_154_Gel in the tumor area, make this treatment highly focused on the cancer cell ablation, reducing the possible side effects that may come from the exposure to high temperatures.

Next, motivated by the cytocompatibility and promising PTT results in vitro (Figure 4), we decided to study the hydrogel in vivo. First, we studied the biocompatibility and degradation of Gel and Mo_154_Gel on healthy C57BL/6J mice. After subcutaneous injection, both hydrogels showed in situ self‐healing and retention under the skin (Figure S7, Supporting Information). The injected hydrogels slowly degraded and became almost invisible after 2 weeks, indicating their biodegradability in vivo. Then, we loaded DOX in the hydrogel and studied the anti‐tumor therapeutic effects of the formulation in vivo, using a murine melanoma model (B16.OVA).^[^ ^67^ ^]^ Here, we assessed five different treatments and animal groups: PBS, Mo_154_Gel, Mo_154_Gel followed by laser irradiation, Mo_154_Gel loaded with DOX, and Mo_154_Gel loaded with DOX followed by laser irradiation, to evaluate the safety profiles and therapeutic effects from PTT, chemotherapy, and combined chemo‐photothermal therapy. The hydrogel injection and laser irradiation were performed on the same day.

**Figure 5:**
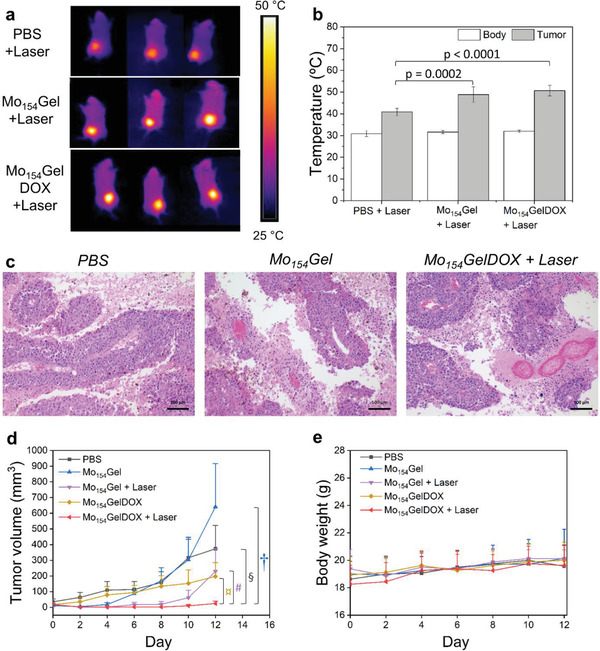
In vivo proof‐of‐concept of the efficacy and safety of Mo_154_Gel on C57BL/6J mice with B16.OVA melanoma. a) Representative IR thermal images of the mice after injection with PBS, Mo_154_Gel, and Mo_154_GelDOX upon NIR irradiation (0.8 W cm^−2^) after 10 min. b) Body and tumor temperature of the mice injected with PBS, Mo_154_Gel, and Mo_154_GelDOX upon 10 min of NIR irradiation (0.8 W cm^−2^). The results were plotted as mean ± standard deviation (*N* = 7). The statistical test was performed by one‐way ANOVA. c) Hematoxylin and eosin (H&E) staining of tumors after the termination of the animal study. The representative images of PBS, Mo_154_Gel, and Mo_154_GelDOX + Laser groups are shown from left to right. Scale bar, 100 µm. d) Tumor growth curve and e) bodyweight of mice treated with PBS, Mo_154_Gel, Mo_154_Gel, and subsequent laser irradiation (0.8 W cm^−2^, 10 min), DOX‐loaded Mo_154_Gel and DOX‐loaded Mo_154_Gel followed by laser irradiation (0.8 W cm^−2^, 10 min). Results are expressed as mean ± standard error of the mean (*N* = 7). The statistical test was performed by two‐way ANOVA followed by Tukey's post‐test. ¤ *p* < 0.0001, # *p* = 0.053, § *p* < 0.0001, † *p* < 0.001, compared with Mo_154_GelDOX + Laser group. The other *p* values of all comparison groups are listed in Table S1, Supporting Information.

First, we evaluated the in vivo hyperthermia after PTT treatment. When only PBS was injected in mice followed by laser irradiation, there was a mild local temperature increase (**Figure** **5**a,b), which was also observed in previous reports.^[^ ^68^, ^69^, ^70^ ^]^ However, when the mice were injected with Mo_154_Gel and exposed to NIR light, we observed a significant temperature increase to 50 °C, which caused rapid cell death due to necrosis and microvascular thrombosis evidenced by histology studies of tumor necrotic areas (Figure 5c).^[^ ^71^ ^]^ This severe temperature increase was mainly limited to the irradiated area, with minimal increase (1 °C) in the body temperature.

Then we characterized the tumor volume increase in all the treatment groups. The tumor growth curves of different formulations are shown in Figure 5d and Figure S8, Supporting Information. The tumor growth profile in mice treated with Mo_154_Gel was similar to the one treated with PBS, suggesting that {Mo_154_} itself or any other component in the hydrogel had no effects on tumor progression. The mice in the group of chemotherapy only (Mo_154_GelDOX) showed slower tumor growth compared with the PBS‐treated group due to the tumor inhibition effect from DOX. The mice receiving PTT only (Mo_154_Gel + Laser) showed significant tumor inhibition right after the treatment (*p* < 0.05 compared with PBS, Mo_154_Gel and Mo_154_GelDOX groups at Day 6, 8, and 10). However, after 1 week, half of the treated mice in Mo_154_Gel + Laser group showed tumor recurrence (Figure S8, Supporting Information). Importantly, the final formulation, Mo_154_GelDOX + Laser, showed the lowest tumor volume among all groups during the entire study period compared with PBS, Mo_154_Gel and Mo_154_GelDOX. The combined chemo‐photothermal therapy showed better efficacy in controlling the tumor growth when compared to each single therapy, with complete tumor eradication in five mice. This is possibly achieved by hyperthermia‐induced tumor ablation and the removal of residual surviving cancer cells by chemotherapy.^[^ ^17^ ^]^

Simultaneously with the evaluation of the therapeutic efficacy of our formulation, we also assessed its safety. As previously discussed, after laser irradiation, the local temperature reached 50 °C in the area irradiated (Figure 5a,b). This high temperature originated local burns in the mice that were treated by honey‐based wound care cream and healed after 4–10 days. Additionally, we also verified that none of the formulations induced body weight loss after the treatment (Figure 5e).

Next, we examined the histology of the major organs (heart, liver, spleen, kidney, and lung) in the representative groups: PBS, Mo_154_Gel, and Mo_154_Gel loaded with DOX followed by laser irradiation (Figure S9, Supporting Information). The tumor inoculation procedure and the treatments did not induce histopathological changes to the studied organs. Furthermore, we analyzed the spleen monocytes of different treatment groups (Figure S10, Supporting Information). No systemic inflammation was found in the tissue, according to the percentage of CD80 and CD86 positive antigen presenting cells and CD8 positive T cells analyzed. These results, along with the bodyweight profile, suggest that Mo_154_Gel based chemo‐photothermal therapy has no systemic toxicity in vivo in the conditions tested.

## 3 Conclusion

In conclusion, we have successfully prepared a self‐healing and injectable hydrogel incorporating both {Mo_154_} and DOX for synergistic photothermal and chemotherapy applications. Besides being a photothermal transducer agent allowing the simultaneous PTT and NIR‐triggered chemotherapy drug release, {Mo_154_} also behaves as a cross‐linker (through electrostatic interactions), taking an active part in the hydrogel structure, and thus, in its mechanical properties. Mo_154_Gel was prepared through a quick and straightforward process, gelating in less than 60 s. It was formed by a dynamic double cross‐linked network that allows the fast re‐formation of the bonds after damage, imparting the hydrogel with great self‐healing and injectability properties. Mo_154_Gel was able to deliver an anticancer drug in a dual laser‐ and pH‐triggered manner. All these advantageous properties of this system were further demonstrated by the efficient ablation of in vivo cancer melanoma without observable systemic toxicity. Therefore, the incorporation of {Mo_154_} in a biocompatible and safe hydrogel paves the way for the use of other POMs in biomedical applications, taking advantage of their diverse properties.

## 4 Experimental Section

The experimental details are reported in the Supporting Information.

## Conflict of Interest

The authors declare no conflict of interest.

## Supporting information

Supporting Information

Supplemental Video 1

## Data Availability

Research data are not shared.
