# Supplementary material for: Dual‐Crosslinked Dynamic Hydrogel Incorporating {Mo154} with pH and NIR Responsiveness for Chemo‐Photothermal Therapy
Source: Adv Mater. 2021 Aug 11;33(40):2007761. doi: 10.1002/adma.202007761 (PMC11468987; doi:10.1002/adma.202007761)
Supplement: Supplementary file 1 — Supporting Information [file ADMA-33-2007761-s002.pdf]

© 2021 Wiley-VCH GmbH

ADVANCED MATERIALS

Supporting Information

for *Adv. Mater.,* DOI: 10.1002/adma.202007761

Dual-Crosslinked Dynamic Hydrogel Incorporating {Mo_{154}} with pH and NIR Responsiveness for Chemo-Photothermal Therapy

Gabriela Guedes, Shiqi Wang, Flavia Fontana, Patrícia Figueiredo, Jere Lindén, Alexandra Correia, Ricardo J. B. Pinto, Sami Hietala, Filipa L. Sousa, and Hélder A. Santos**

Copyright WILEY-VCH Verlag GmbH & Co. KGaA, 69469 Weinheim, Germany, 2018.

Supporting Information

Dual-crosslinked dynamic hydrogel incorporating {Mo_{154}} with pH and NIR responsiveness for chemo-photothermal therapy

Gabriela Guedes,[1] Shiqi Wang,[1] Flavia Fontana,[1] Patrícia Figueiredo, Jere Lindén, Alexandra Correia, Ricardo J. B. Pinto, Sami Hietala, Filipa L. Sousa, Hélder A. Santos**

G. Guedes, Dr. S. Wang, Dr. F. Fontana, Dr. P. Figueiredo, A. Correia, Prof. H. A. Santos
Drug Research Program
Division of Pharmaceutical Chemistry and Technology
Faculty of Pharmacy
University of Helsinki
FI-00014 Helsinki, Finland
E-mail: shiqi.wang@helsinki.fi; helder.santos@helsinki.fi

G. Guedes, Dr. R. J. B. Pinto, Dr. F. L. Sousa
Chemistry Department
University of Aveiro
Campus Universitário de Santiago
3810-193 Aveiro, Portugal

G. Guedes, Dr. R. J. B. Pinto, Dr. F. L. Sousa
CICECO-Aveiro Institute of Materials
University of Aveiro
Campus Universitário de Santiago
3810-193 Aveiro, Portugal

Dr. J. Lindén
Faculty of Veterinary Medicine
Finnish Centre for Laboratory Animal Pathology (FCLAP) / HiLIFE
University of Helsinki
FI-00014 Helsinki, Finland

Dr. S. Hietala
Department of Chemistry
University of Helsinki
FI-00014 Helsinki, Finland

Prof. H. A. Santos
Helsinki Institute of Life Science (HiLIFE)
University of Helsinki
FI-00014 Helsinki, Finland

[1]These authors contributed equally to the work

Experimental Section

Materials: **{Mo154}** Sodium dithionite ($Na_2S_2O_4$), sodium molybdate (Na_2MoO_4•$2H_2O$), HCl (1 M) were obtained from Alfa Aesar (Germany). **Polymers:** ammonium persulfate (APS, ≥98.0 %); chitosan (CS, medium molecular weight, 75-85% deacetylated); deuterium chloride solution (35 % (w/w) in D_2O, ≥99 atom % D), deuterium oxide (99.9 atom % D), dimethyl sulfoxide-d_6 (99.9 atom % D, contains 0.03 % (v/v) TMS); N,N'-dicyclohexylcarbodiimide (DCC, ≥99 %), 4-dimethylaminopyridine (DMAP, ≥99 %), diethyl ether (contains BHT as inhibitor, ≥99.8 %), 4-formylbenzoic acid (97 %), glacial acetic acid (≥99.8 %), N-isopropylacrylamide (NIPAAm, 97%) and tetrahydrofuran (THF, ≥99.9 %) were obtained from Sigma-Aldrich (Finland). Dialysis bag (Spectra/Por® 6 Dialysis bag Membrane Pre-wetted RC tubing MWCo: 50 kDa) purchased from Spectra (USA). Polyethylene glycol (PEG, 4000 Da) was acquired from Fluka® (Finland). Filter paper (qualitative 4) was purchased from Whatman® (Finland). **Release studies:** Doxorubicin hydrochloride (>95.0 %) was obtained from Tokyo Chemical Industry Co. LTD (Finland). Hank's balanced salt solution (HBSS, 10×) was purchased from Life Technologies (USA). 2-(N-morpholino)-ethanesulfonic acid (MES); and 4-(2-hydroxyethyl)-1-piperazineethanesulfonic acid (HEPES) were purchased from Sigma-Aldrich (Finland). **Cell culture:** Dulbecco's Modified Eagle Medium (DMEM), Roswell Park Memorial Institute (RPMI) 1640, and fetal bovine serum (FBS) were purchased from Life Technologies Gibco®, USA. Non-essential amino acids (NEAA), L-glutamine 200 mM, penicillin (100 IU mL^{-1}), streptomycin (100 mg mL^{-1}) and trypsin (2.5%) were purchased from HyClone™, GE Healthcare Lifesciences (Logan, UT, USA). Triton X-100 was purchased from Merck Millipore (Darmstadt, Germany).

Synthesis of {Mo$_{154}$}: $Na_{15}[Mo^{VI}_{126}Mo^{V}_{28}O_{462}H_{14}(H_2O)_{70}]_{0.5}[Mo^{VI}_{124}Mo^{V}_{28}O_{457}H_{14}(H_2O)_{68}]_{0.5}]$ ·ca.400H_2O ≡ **{Mo$_{154}$}** (the second constituent has one {Mo_2} group missing. Complete Mo_{154} cluster anions and defective ones at a ratio of 1:1 coexist in the crystaline product.) was

prepared following the method described elsewhere.[1] Briefly, $Na_2S_2O_4$ (0.2 g, 1.15 mmol) was added to an aqueous solution of $Na_2MoO_4 \cdot 2H_2O$ (3.0 g, 12.4 mmol) prepared in 10 mL of water. Next, under continuous stirring, 30 mL of HCl (1 M) were added. The solution was allowed to stir for 10 min in an open Erlenmeyer flask. Afterwards, the solution was stored in a closed flask for five days. Then, the precipitated blue crystals were removed by filtration and washed with cold water, and dried in a desiccator at room temperature.

Synthesis of poly(N-isopropylacrylamide) functionalized chitosan (CS-g-PNIPAAm): CS-g-PNIPAAm was prepared by free radical polymerization adapting the method previously reported by Duan et al.[2] The co-polymerization reaction was carried out in a round bottom flask equipped with a magnetic stirrer and capped with a septum. After dissolving 1.0 g of CS in 200 mL of acetic acid (0.6 v-%), the chitosan solution was heated to 80 ºC under nitrogen. Then, 3 mL of APS (1×10^{-2} mol L^{-1}) were added to the solution and stirred for 10 min. Finally, 6 mL of NIPAAm solution (2.9 mol L^{-1}) were added, and the solution stirred for 3 h. The obtained product was dialyzed in Milli-Q water for five days and finally freeze-dried. The product obtained had a mass of 0.97 g with a yield of 65%. The polymer was characterized by ^{1}H-NMR with an Avance III 400 MHz NMR spectrometer (Bruker, Switzerland) to confirm the structure (**Figure S1**). By integrating the characteristic peaks from polyNIPAAm (at 1.10 ppm, 6H) and CS (at 3.14 ppm, 1H), it was found that there were on average 3.9 NIPAAm monomers per glucosamine unit on CS, which means the weight percentage of PNIPAAm and CS in the final polymer is around 73% and 27%, respectively. The thermo-responsiveness and the LCST of CS-g-PNIPAAm were studied by DSC using a Mettler Toledo DSC826e with a flow rate of 1 ºC min^{-1} under a nitrogen atmosphere (**Figure S4**).

Synthesis of di-functionalized PEG (DF-PEG): PEG was functionalized with benzaldehyde groups adapting the procedure previously described by Zhang et al.[3] Briefly, PEG4000 (3.26 g, 0.81 mmol) was dissolved in 50 ml of THF, followed by the addition of 4-formylbenzoic acid (0.49 g, 3.26 mmol), DMAP (0.025 g, 0.20 mmol), and DCC (0.84 g, 4.07 mmol). The

mixture was allowed to react under magnetic stirring overnight, and then the white precipitate was removed by filtration. The polymer solution was purified by precipitation in diethyl ether three times, and then the polymer was obtained as a white solid. After drying in an oven (Memmert GmbH) at 37 ºC overnight, the obtained product had a mass of 2.45 g with a yield of 75%. The polymer was characterized by ^{1}H-NMR to confirm the structure (**Figure S2**). By integrating the characteristic peaks from the conjugated benzaldehyde (8-8.2 ppm, 4H) and PEG (at 3.38-3.87 ppm, ~364 H from one PEG 4000 chain), it was estimated that 79.4% of the PEG molecules were successfully modified with benzaldehyde groups at both ends.

***Synthesis of Mo_{154}Gel*:** A 2 wt-% CS-g-PNIPAAm solution was obtained by dissolving the polymer in 0.01 v-% acetic acid aqueous solution. A 33 wt-% DF-PEG solution was prepared by dissolving 500 mg of the polymer in 1 mL of Milli-Q water. A solution containing 1.1 wt-% of {Mo_{154}} and 44 wt-% PEG was prepared by dissolving 20 mg of POM and 800 mg of PEG in 1 mL of Milli-Q water. Furthermore, for the blank hydrogels, it was prepared a 44 wt-% solution of PEG by dissolving 800 mg of PEG in 1 mL of Milli-Q water.

Briefly, the {Mo_{154}}+PEG solution (25 μL) was added to the DF-PEG solution (50 μL) and the CS-g-PNIPAAm solution (225 μL). Finally, all the components were mixed for 30 s using vortex, and within less than 1 min the hydrogel was formed.

Gel was prepared in the same way, but instead of adding {Mo_{154}}+PEG solution, it was added only a PEG solution. After preparation, the Mo_{154}Gel and Gel were freeze-dried before characterization on a Bruker Vertex 70 spectrometer, using an ATR–FTIR instrument. The FTIR spectra were recorded in the range of 4000–400 cm^{-1} with a resolution of 2 cm^{-1} using OPUS 8.1 software. Raman spectroscopy of Mo_{154}Gel and {Mo_{154}} was also recorded from 1750 to 100 cm^{-1}, using a spectrophotometer Raman-FT Bruker RFS/100S. The UV-Vis absorption spectra of a {Mo_{154}} aqueous solution, Gel and Mo_{154}Gel were recorded using a Varioskan™ LUX multimode microplate reader between 400 to 1000 nm.

Energy-dispersive X-ray spectroscopy (EDS)-mapping micrographs were acquired in a FEG-SEM Hitachi SU-70 operated at 15 kV with EDS Bruker QUANTAX 400 detector. The Mo_{154}Gel samples were prepared by freeze-drying, as described above, and placed in an aluminum support with double-sided carbon tape and coated with carbon using an EMITECH K950 coating system beforeanalysis.

Rheological analysis: The rheological behavior of both Mo_{154}Gel and Gel were measured by shear rheometry using a TA Instruments AR2000 rheometer with a 20 mm diameter stainless steel plate-and-plate (parallel plate) geometry. Five different test methods were employed: oscillatory time sweep, strain sweep, frequency sweep, temperature ramp sweep and flow sweep. For the time sweep test, all the components of the hydrogel were added to the down plate, and the test was initiated right after. The strain was kept at 1% with an angular frequency of 1 Hz, at 25 ºC, for 10 min. The strain sweep test was performed at 25 ºC and the moduli recorded from 0.9% to 1000% strain at a constant frequency of 1 Hz. The hydrogels were then subjected to a frequency sweep at a constant strain (1%) within the linear viscoelastic range at 25 ºC. The G' and G'' values were recorded with the increase of the oscillatory frequency from 20 to 0.1 rad/s. The temperature ramp sweep was performed on Mo_{154}Gel from 15 to 50 °C (3 °C / min) at a constant frequency (1 Hz). The flow properties of the Mo_{154}Gel were studied by ramping the shear rate from 1.0 to 158 s^{-1} at 25 ºC.

Furthermore, to study the self-healing behavior of the prepared formulation, step strain measurements were also performed. The strain was changed from 1% to 1000%, keeping the angular frequency (1 Hz) and the temperature (25 ºC) constant.

To further study the self-healing of the prepared material, a freshly prepared hydrogel was placed in a glass coverslip and a hole with ~0.8 cm in diameter was made in the center of the hydrogel. The closure of the hole was recorded, and the time passed until the complete recovery of the hydrogel counted.

The injectability of the developed hydrogel was studied by extrusion of the hydrogel through a needle (Terumo® AGANI 22G×5/8, 0.7×40 mm). Initially, the hydrogel was loaded into a 10 mL syringe (Soft-Ject®, from Henke Sass Wolf, Germany) before gelation, and after 5 min extruded through a needle into a glass lamella to write the letters "UH".

Photothermal conversion studies: The photothermal conversion of the prepared hydrogel was studied using an 808 nm NIR laser (RLTMDL-808-2W, Roithner Lasertechnik GmbH, Austria). Mo_{154}Gel was prepared in a 2 mL vial and the laser was irradiated from the top of the vial. The temperature of the hydrogel was monitored by an IR camera (FLIR C2, Sweden) from the side of the vial. The images and the temperature data were processed by FLIR Tools Software.

To study the increase of the temperature variation of with the concentration of photothermal agent {Mo_{154}}, hydrogels with different POM concentrations were prepared by changing the initial concentration of the {Mo154}, to have the final concentrations of 0, 0.023, 0.046, 0.092 wt-%. To study the effects of laser power, hydrogel containing 0.092 wt-% {Mo_{154}} was irradiated by laser at 0.4, 0.6 and 0.8 W cm^{-2}, respectively. Gel without {Mo_{154}} was also irradiated at the same power as a control. To study the photothermal stability of Mo_{154}Gel, hydrogel containing 0.092 wt-% {Mo_{154}} was irradiated at 0.8 W cm^{-2} for 10 min, and then the laser was shut down for 20 min, for the hydrogel to cool down to room temperature. This ON/OFF laser irradiation procedure was repeated for three cycles.

***In vitro release study*:** the hydrogels were prepared as previously described with the further addition of 25 μL of DOX (1 mg mL^{-1}). The release experiments were performed by soaking the hydrogels in 1.5 mL of the appropriate buffer (HBSS–HEPES (pH 7.4) or HBSS–MES (pH 5.5 or 6.2)) at 37 ºC. In each time point (0, 10, 30, 90, 120, 180, 240, and 300 min), 10 μL of the release medium was removed and substituted by the same volume of fresh buffer. To study the laser-triggered release, a further release study was performed: firstly,

DOX@Mo_{154}Gel were allowed to equilibrate with the buffer medium (HBSS–HEPES, pH 7.4) for 60 min at 37 ºC, and then they were irradiated for 10 min with a laser (0.8 W cm^{-2}) and left without laser irradiation for another 10 min; this procedure was repeated in three cycles at pH 6.2 and 7.4. For comparison, three DOX@Mo_{154}Gel were soaked in the same conditions, but without any laser irradiation (pH 7.4 at 37 ºC). The laser-triggered release sample was taken every 10 min until the three cycles finished. All the aliquots removed were diluted 10 times, centrifuged (5 min, 9000 rpm), and 80 μL were removed from the supernatant to a plate posteriorly analyzed by fluorescence (λ_{ex} = 470 nm; λ_{em} = 585 nm). For each condition, the analysis was done in triplicate. The concentration of DOX release in each time point was determined using a calibration line.

***Cytotoxicity studies*:** B16. OVA murine melanoma cells were cultured in RPMI supplemented with 10% of FBS, 1% of L-glutamine, 1% of NEAA, penicillin (100 IU mL^{-1}), streptomycin (100 mg mL^{-1}) and 50 mg mL^{-1} of G418 (ThermoFisher, USA). M21 human melanoma cells and primary human fibroblast cells were cultured in DMEM with 4.5 g L^{-1} of glucose, supplemented with 10% of FBS, 1% of L-glutamine, 1% of NEAA, penicillin (100 IU mL^{-1}) and streptomycin (100 mg mL^{-1}). The cells were cultured in the 5% CO_2-incubator at 37 °C, and 95% relative humidity. Prior to each test, the cells were passaged using 0.25% (v/v) trypsin in PBS.

For the cytotoxicity study of free Mo_{154}, primary human fibroblast cells, B16.OVA or M21 cells were seeded in a 96-wellplate at 1 × 10^4 cells per well. After overnight attachment, the Mo_{154} suspended in corresponding cell culture medium were added at concentrations of 0.2, 0.46, 0.92, 1.84 and 2.76 mg mL^{-1}. Cell culture medium without any tested materials were used as controls. After 4, 24, 48 and 72 h, the cell viability was measured using a CellTiter-Glo® luminescent assay and the luminescence obtained from a Varioskan™ LUX multimode microplate reader. All samples were tested in 4 replicates. The significance was analyzed by

one-way ANOVA. The levels of significant differences were set at probabilities of *P < 0.05, **P < 0.01, and ***P < 0.001.

For the cytotoxicity study of Gel and Mo_{154}Gel, primary human fibroblast cells, B16.OVA or M21 cells were seeded in a 24-well Transwell® plate at 5 × 10^4 cells per well. The cells were allowed to attach overnight. Then the freshly prepared Gel or Mo_{154}Gel (300 μL final volume), as described above, were added to the upper chamber of the Transwell and waited for gelation. The hydrogels in the upper chamber of the Transwell were added back to the cell culture medium. Triton X-100 solution (1% v/v in cell culture medium), and cell culture medium without any tested materials were used as negative and positive controls, respectively. After 4, 24, 48 and 72 h, the cell viability was measured using a CellTiter-Glo® luminescent assay. After cell lysis, the CellTiter-Glo® assay was performed in a 96-well plate, using the Varioskan™ LUX multimode microplate reader. All samples were tested in triplicates. The significance was analyzed by one-way ANOVA. The levels of significant differences were set at probabilities of *P < 0.05, **P < 0.01, and ***P < 0.001.

***In vitro PTT*:** M21 cells were seeded in a 96-well plate at a density of 1 × 10^4 cells per well one day piror to the experiment. Then the cell culture medium in each well were replaced by 100 μL HBSS–HEPES buffer, and 50 μL Mo_{154}Gel were injected to the wells. Cells without Mo_{154}Gel injections were used as controls. The cells receiving in vitro PTT treatment were exposed to NIR laser (808 nm) at 0.8 W cm^{-2} for 10 min. After the laser exposure, the cell viability was measured using a CellTiter-Glo® luminescent assay according to the manufacture's protocol. The luminescence intensity was obtained from a Varioskan™ LUX multimode microplate reader.

Animal experiments: C57BL/6J female mice (4-6 weeks) were obtained from Envigo. The mice were housed in individually ventilated cages with ad libitum feeding. During each procedure (imaging, injection and laser treatment), mice were anesthetized by isoflurane inhalation. All the procedures used were reviewed and approved by the Experimental Animal

Committee of the University of Helsinki and the Provincial Government of Southern Finland license number ESAVI/11895/2019.

For the hydrogel degradation studies, 200 μL of PBS, Gel or Mo_{154}Gel were injected subcutaneously on the lower back area of healthy mice (4 mice per group). The mice were checked and imaged regularly after the injection (3h, and daily from Day1 to Day 14). After the hydrogels became almost invisible, the mice were euthanized (on Day 14), and the skin of injected area was opened to check if any hydrogel residual was retained.

For tumor inoculation, 2.5×10^5 of B16.OVA cells resuspended in 100 μL of non-supplemented RPMI were injected subcutaneously on the lower back area. After 16 days, the mice were randomized into 5 groups (8 mice per group) with different formulations injected intratumorally. Group 1 was injected with 100 μL of PBS, while Group 2 and 3 were injected with 100 μL of Mo_{154}Gel. Group 4 and 5 were injected with 100 μL Mo_{154}Gel containing DOX (8.5 μg). After injection, Group 3 and 5 were exposed to NIR laser (808 nm) at 0.8 W cm^{-2} for 10 min and the temperature after treatment was recorded using an IR camera (FLIR C2, Sweden). The tumor size and body weight of the mice was measured every 2 days since the treatment, and the mice were euthanized 18 days after the treatment.

For histological analysis, the major organs from each group (heart, liver, spleen, lung and kidney) and tumors were sampled and fixed with 4% paraformaldehyde for 48 h at 4 °C, and finally transferred to 70% ethanol solution. The organs and tumors were embedded in paraffin, sectioned at 4 µm and stained with hematoxylin and eosin (H&E).

For immunological analysis, the mice spleens were collected and a uniform single-cell suspension was obtained by gently grinding the tissue on a 70 μm cell strainer. The samples were frozen at –80 °C in FBS supplemented with 10% of DMSO until analysis. On the day of analysis, the samples were thawed quickly in a 37 °C waterbath and washed once with PBS. The samples were blocked with murine Fc block CD16/32 (Biolegend, USA) from unspecific

staining and then stained according to two different panels of antibodies on ice. The CD3e-FITC, CD4-PerCP and CD8-APC (all from for Biolegend, USA) were used for T cells, while CD11c-FITC (BD Biosciences, USA), CD80-PE (Biolegend, USA) and CD86-APC (Biolegend, USA) were used for antigen presenting cells. The cells were subsequently washed twice with PBS to removed the unstained antibodies, and analyzed by Accuri C6 Flow Cytometer. The data acquired were processed using FlowJo software.

Statistical analysis: The statistical significance analysis was analyzed using OriginPro 2018 software. The sample size and test method details description can be found in each experimental section separately.

Supplementary Figures

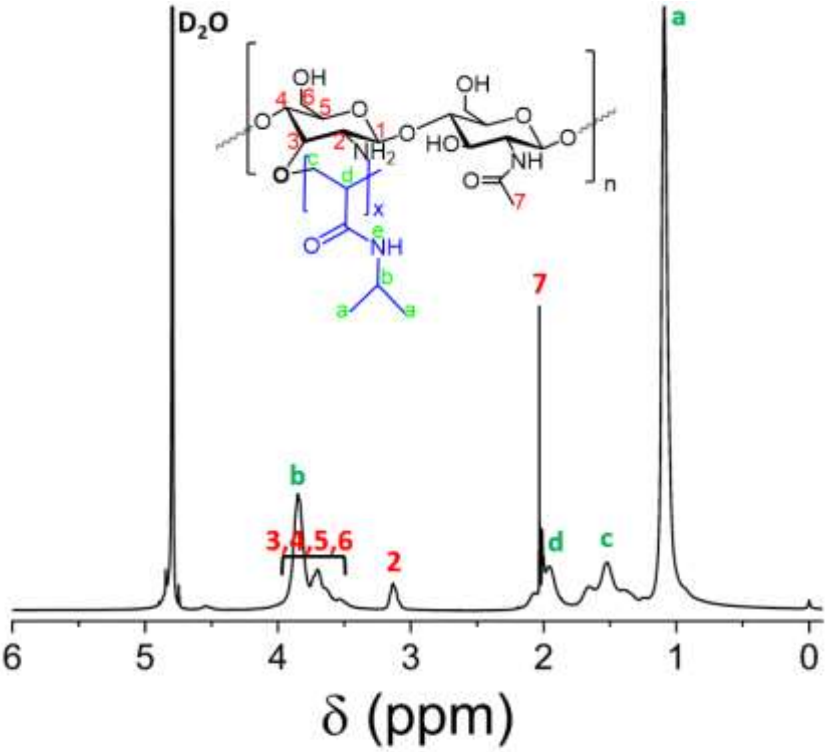

Figure S1. ^{1}H NMR spectrum of CS-g-PNIPAAm in 1% of deuterium chloride. In the inset is presented the structural formula of CS-g-PNIPAAm copolymer. The letters from a to d represent the different protons in the NIPAAm, and the numbers from 1 to 7 represent the protons on the sugar unit of CS-g-PNIPAAm.

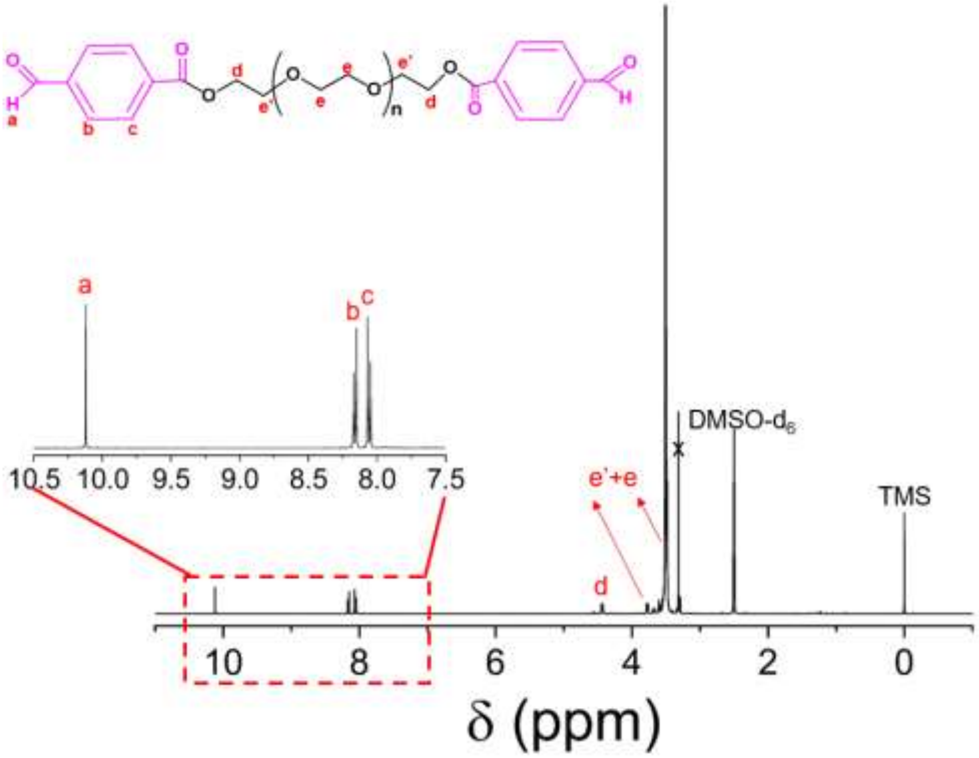

Figure S2. ^{1}H NMR spectrum of DF-PEG in DMSO-d_6. In the inset is presented the structural formula of DF-PEG. The letters from a to e represent the distinct protons in the DF-PEG molecule.

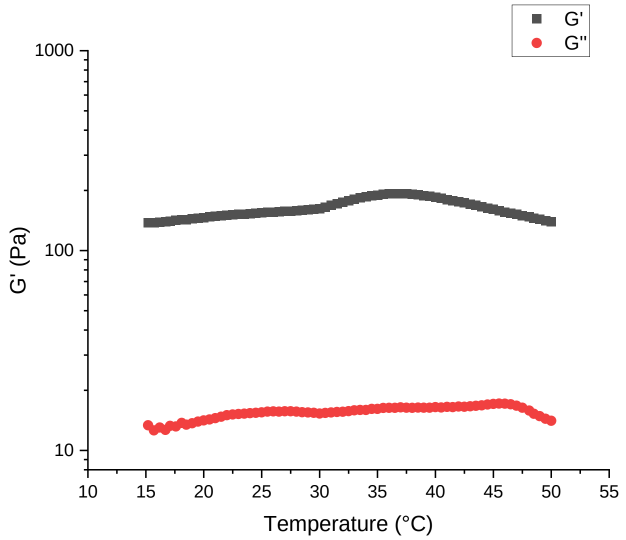

Figure S3. The temperature ramp study of Mo_{154}Gel from 15 to 50 °C (3 °C / min), at a constant frequency (1 Hz) and oscillation stress (6.37 Pa).

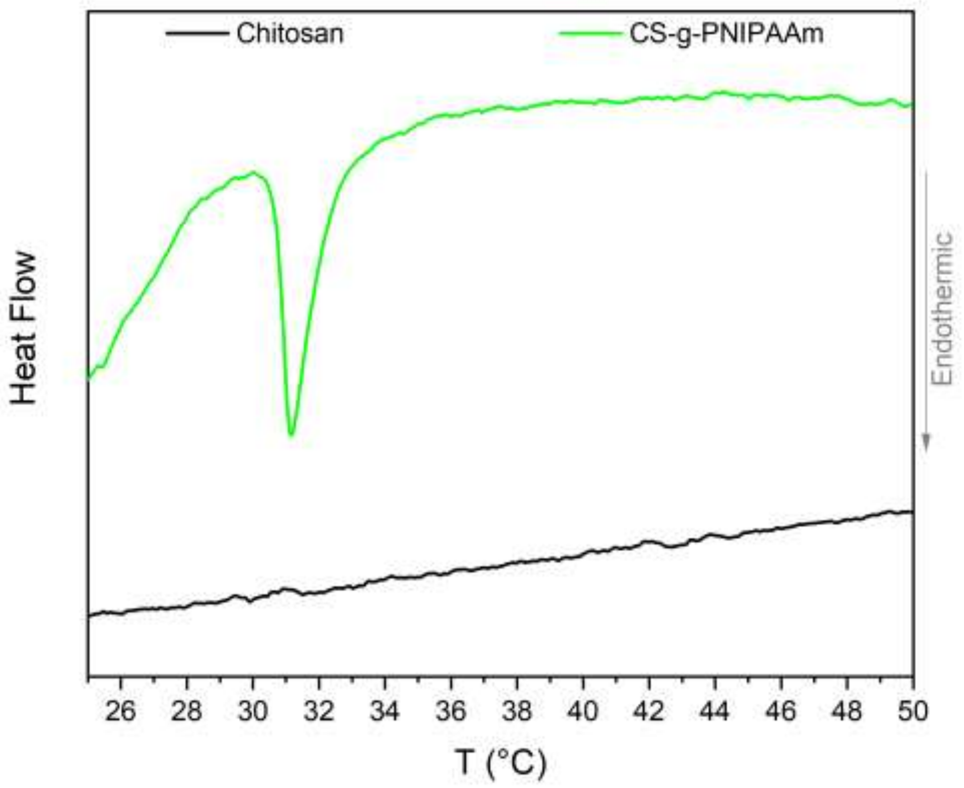

Figure S4. Heat flow versus temperature of CS (2.0 wt-%) and CS-g-PNIPAAm solutions (1.5 wt-%) measured by DSC.

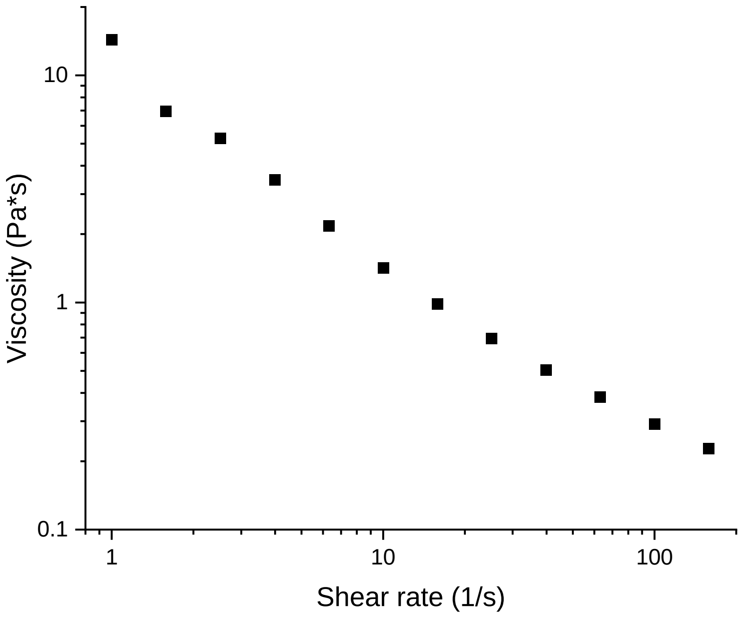

Figure S5. The flow sweep of Mo_{154}Gel at 25 °C.

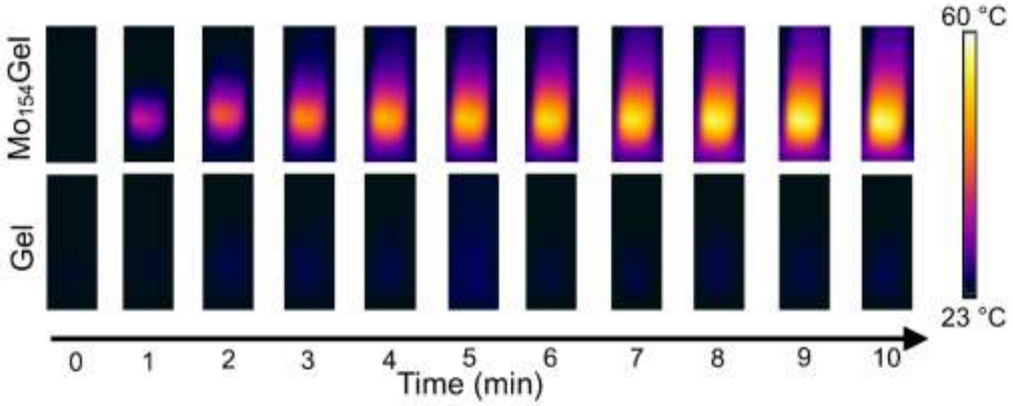

Figure S6. IR thermal images showing the temporal evolution of the temperature of the Mo_{154}Gel and Gel upon NIR irradiation (0.8 W cm^{-2}).

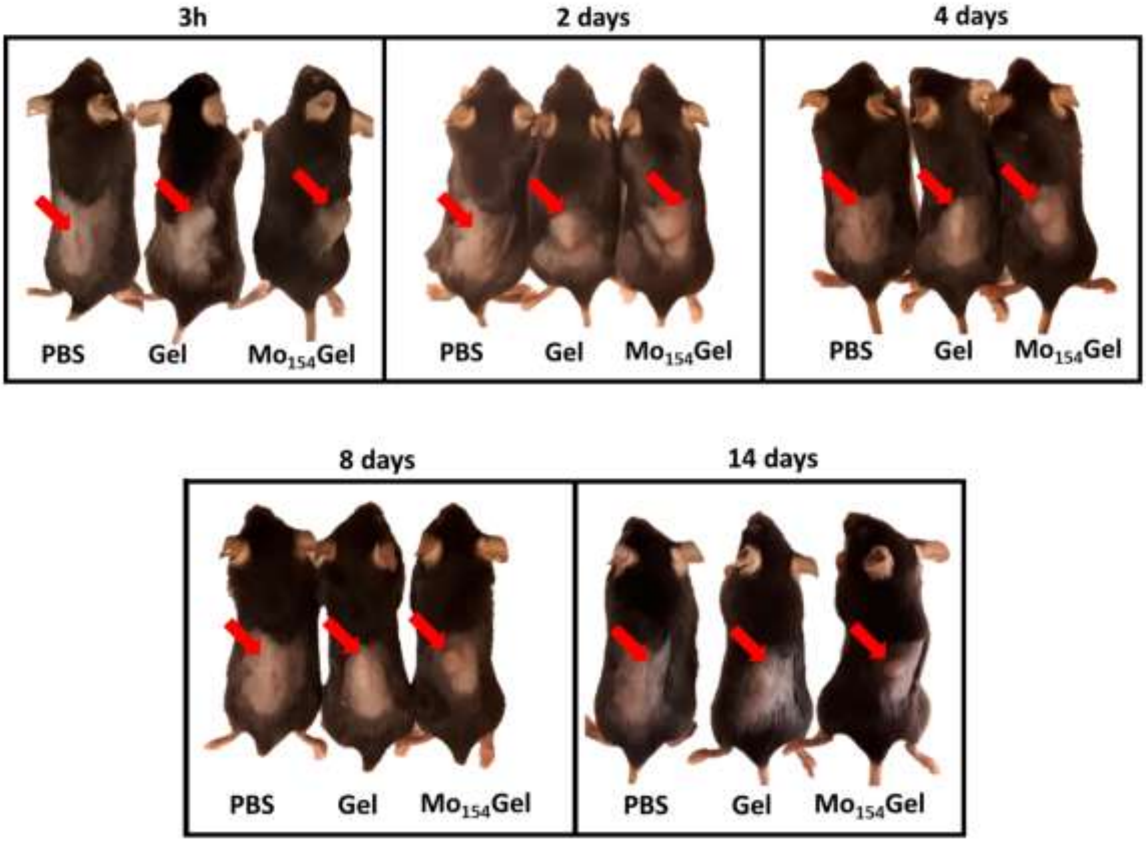

Figure S7. Hydrogel degradation in vivo in healthy C57BL/6J mice. The red arrows indicate the location of the injection.

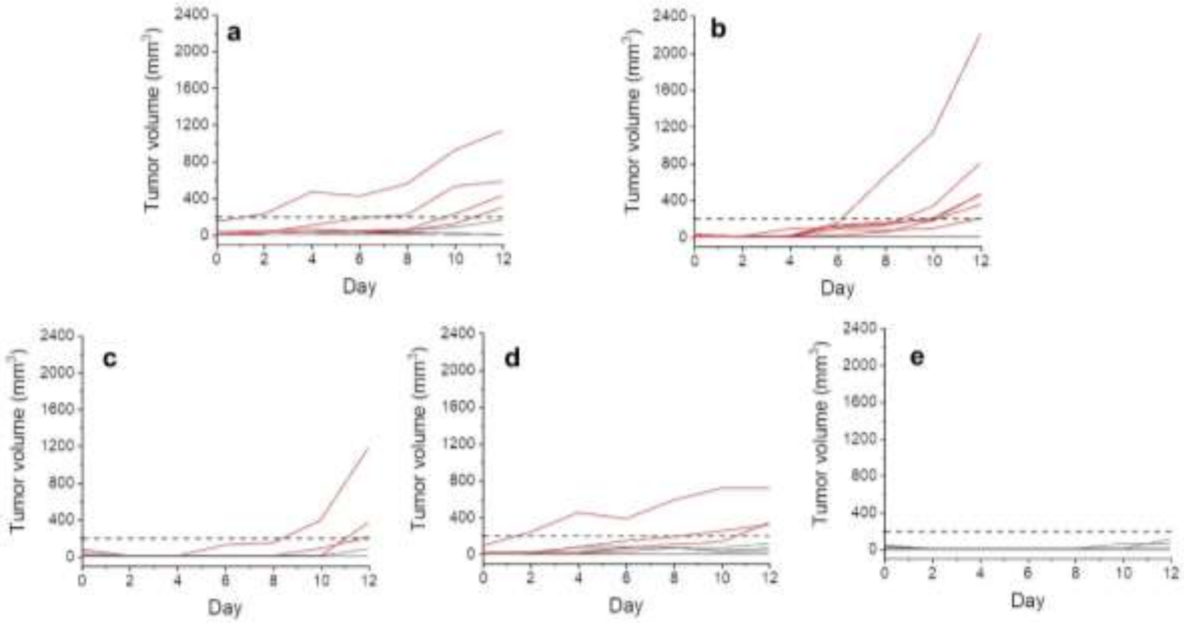

Figure S8. Individual tumor growth curve of mice treated with (a) PBS, (b) Mo_{154}Gel, (c) Mo_{154}Gel and subsequent laser irradiation, (d) DOX loaded Mo_{154}Gel and (e) DOX loaded Mo_{154}Gel followed by laser irradiation. The dash lines represent a tumor volume of 200 mm^3. The growth curves with final volume higher than 200 mm^3 were plotted in red, while those lower than 200 mm^3 were plotted in grey.

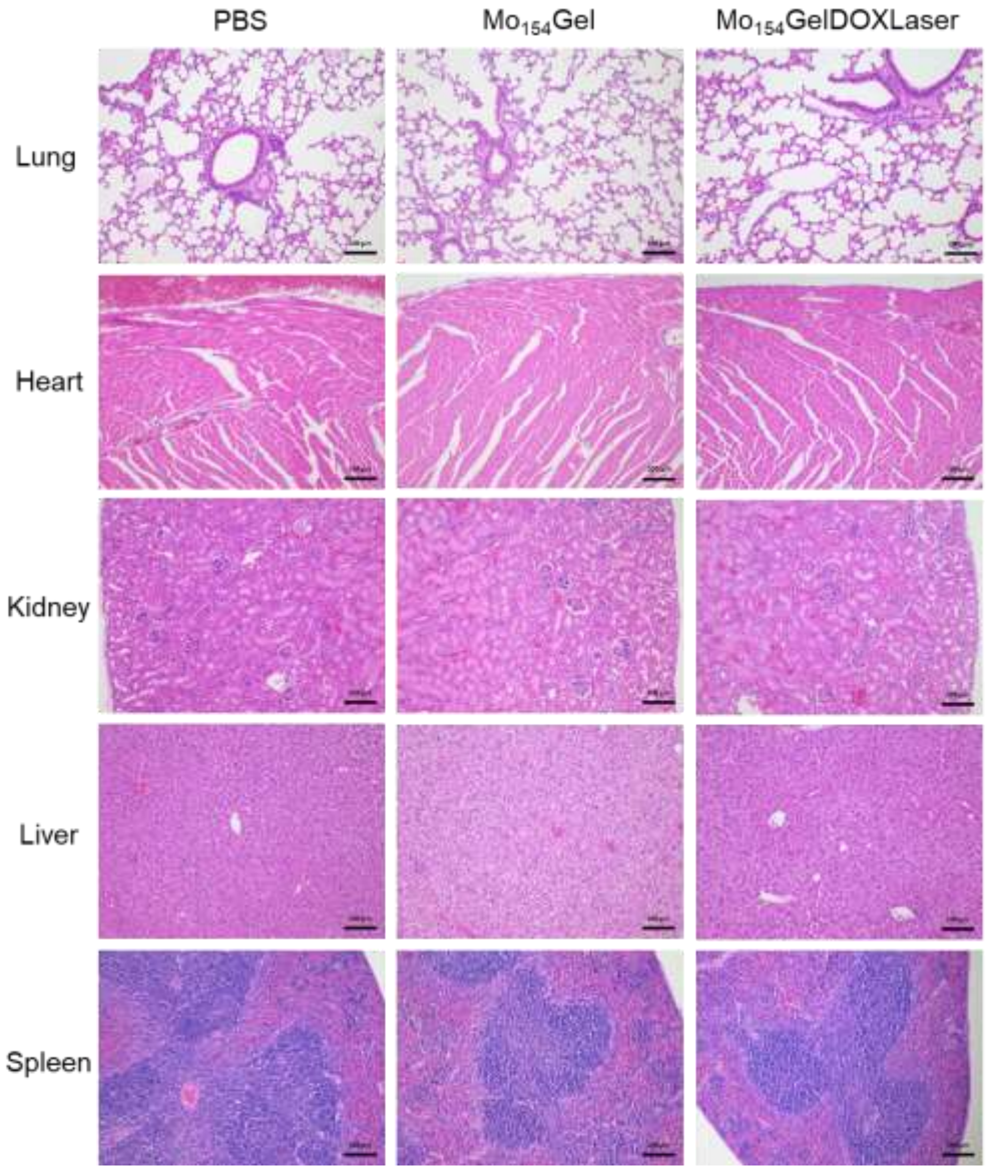

Figure S9. Representative H&E staining images of the major peripheral organs of mice subcutaneously injected with PBS, Mo_{154}Gel and Mo_{154}Gel loaded with DOX followed by laser irradiation. Scale bars are 100 µm.

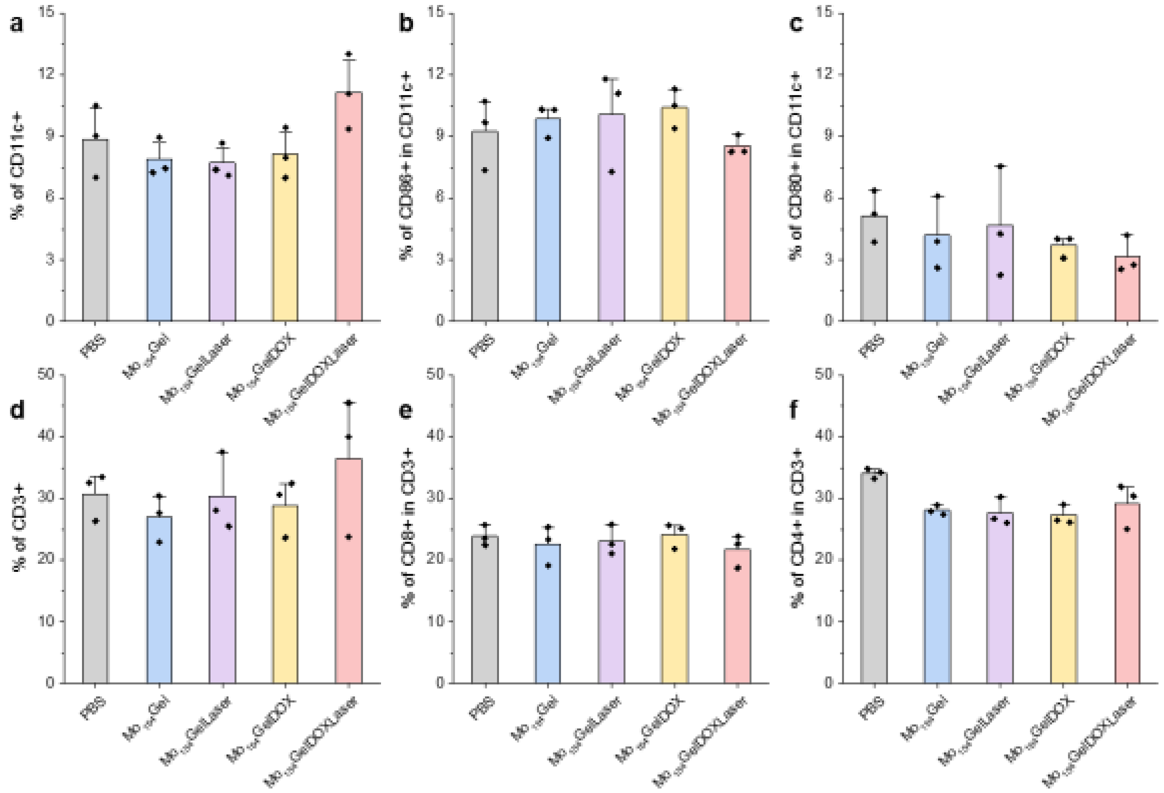

Figure S10. Flow cytometry results of the spleen monocytes of mice treated with PBS, Mo_{154}Gel, Mo_{154}Gel and subsequent laser irradiation, DOX loaded Mo_{154}Gel and DOX loaded Mo_{154}Gel followed by laser irradiation. (a) Percentage of antigen presenting cells in the spleen, (b, c) Maturation status of antigen presenting cells in the spleen, analyzed by the percentage of cells positive for the co-stimulatory signals CD80 and CD86. (d) Percentage of T cells in the spleen. (e, f) Analysis of the T cell population in the spleen divided into CD8+ cytotoxic cells and CD4+ helper cells . Original individual data points were plotted as mean ± standard error of the mean (N=3).

Table S1. *P*-values of all the relevant comparisons between groups in Figure 5d. The statistical test was performed by two-way ANOVA followed by Tukey's post-test.

	PBS	$Mo_{154}Gel$	$Mo_{154}Gel$+Laser	$Mo_{154}GelDOX$	$Mo_{154}GelDOX$+Laser
PBS		0.67	0.023	0.38	3.6×10^{-5}
$Mo_{154}Gel$	0.67		0.025	0.26	4.9×10^{-4}
$Mo_{154}Gel$+Laser	0.023	0.025		0.098	0.053
$Mo_{154}GelDOX$	0.38	0.26	0.098		5.7×10^{-5}
$Mo_{154}GelDOX$+Laser	3.6×10^{-5}	4.9×10^{-4}	0.053	5.7×10^{-5}	

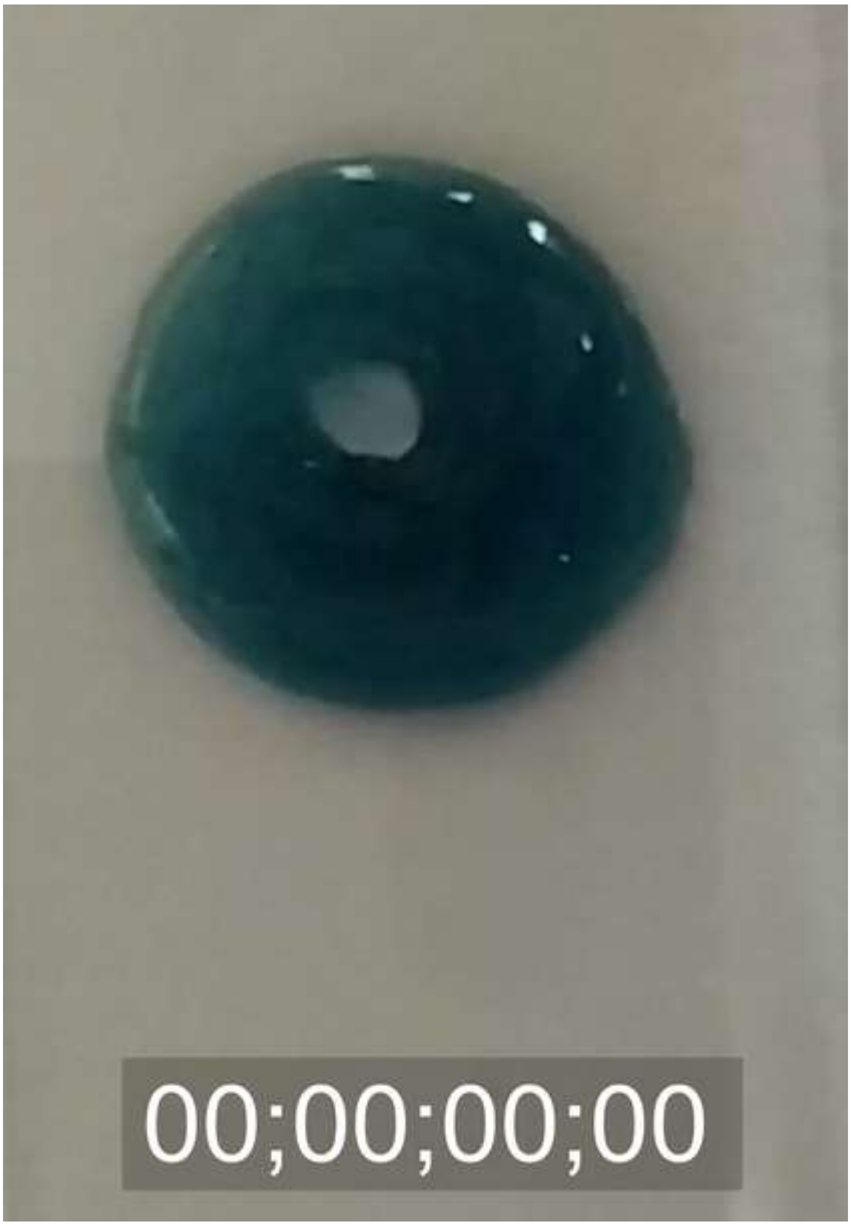

Video S1. Video showing the fast self-healing of Mo_{154}Gel. A hole with around 0.8 cm of diameter completely healed in less than 4 min.

References of supporting information

[1] S. K. Das, E. Krickemeyer, C. Kuhlmann, M. H. Dickman, M. T. Pope, Inorganic Synthesis, John Wiley & Sons, Inc., Hoboken, NJ, USA, 1998.
[2] C. Duan, D. Zhang, F. Wang, D. Zheng, L. Jia, F. Feng, Y. Liu, Y. Wang, K. Tian, F. Wang, Q. Zhang, *Int. J. Pharm.* **2011**, *409*, 252.
[3] Y. Zhang, L. Tao, S. Li, Y. Wei, *Biomacromolecules* **2011**, *12*, 2894.
